# Bioactive Coatings Based on Nanostructured TiO_2_ Modified with Noble Metal Nanoparticles and Lysozyme for Ti Dental Implants

**DOI:** 10.3390/nano12183186

**Published:** 2022-09-14

**Authors:** Emilian Chifor, Ion Bordeianu, Crina Anastasescu, Jose Maria Calderon-Moreno, Veronica Bratan, Diana-Ioana Eftemie, Mihai Anastasescu, Silviu Preda, Gabriel Plavan, Diana Pelinescu, Robertina Ionescu, Ileana Stoica, Maria Zaharescu, Ioan Balint

**Affiliations:** 1 Faculty of Medicine of the Ovidius University, Aleea Universitatii nr.1, 900470 Constanţa, Romania; 2 “Strungareata” SRL, Strada Garii nr. 24, 800217 Galati, Romania; 3 “Ilie Murgulescu” Institute of Physical Chemistry of the Romanian Academy, 202 Spl. Independentei, 060021 Bucharest, Romania; 4 Faculty of Biology, “Alexandru Ioan Cuza” University, 700505 Iasi, Romania; 5 Faculty of Biology, Intrarea Portocalilor 1-3, Sector 5, 060101 Bucharest, Romania

**Keywords:** TiO_2_ coatings for titanium implant, noble metal nanoparticles, photosensitive materials, antibacterial activity, hybrid materials, biocatalytic activity of lysozyme

## Abstract

This work presents the synthesis of nanostructured TiO_2_ modified with noble metal nanoparticles (Au, Ag) and lysozyme and coated on titanium foil. Moreover, the specific structural and functional properties of the resulting inorganic and hybrid materials were explored. The purpose of this study was to identify the key parameters for developing engineered coatings on titanium foil appropriate for efficient dental implants with intrinsic antibacterial activity. TiO_2_ nanoparticles obtained using the sol–gel method were deposited on Ti foil and modified with Au/Ag nanoparticles. Morphological and structural investigations (scanning electron and atomic force microscopies, X-ray diffraction, photoluminescence, and UV–Vis spectroscopies) were carried out for the characterization of the resulting inorganic coatings. In order to modify their antibacterial activity, which is essential for safe dental implants, the following aspects were investigated: (a) singlet oxygen (^1^O_2_) generation by inorganic coatings exposed to visible light irradiation; (b) the antibacterial behavior emphasized by titania-based coatings deposited on titanium foil (TiO_2_/Ti foil; Au–TiO_2_/Ti foil, Ag–TiO_2_/Ti foil); (c) the lysozyme bioactivity on the microbial substrate *(Micrococcus lysodeicticus*) after its adsorption on inorganic surfaces (Lys/TiO_2_/Ti foil; Lys/Au–TiO_2_/Ti foil, Lys/Ag–TiO_2_/Ti foil); (d) the enzymatic activity of the above-mentioned hybrids materials for the hydrolysis reaction of a synthetic organic substrate usually used for monitoring the lysozyme biocatalytic activity, namely, 4-Methylumbelliferyl β-D-N,N′,N″-triacetylchitotrioside [4-MU-β- (GlcNAc)_3_]. This was evaluated by identifying the presence of a fluorescent reaction product, 7-hydroxy-4-metyl coumarin (4-methylumbelliferone).

## 1. Introduction

Titanium-based materials are commonly used for biomedical applications, especially in the dentistry and orthopedic fields, as they are characterized by a wide spectrum of morphological, compositional, and functional parameters [1,2,3]. The intensive development of innovative and valuable dental implants relies on certain prerequisite features and standards of the envisaged materials, such as appropriate mechanical resistance, biocompatibility [1], and the ability to promote osseointegration [2]. Moreover, numerous research studies and studies of medical technologies focus on materials that have their own intrinsic antibacterial capacities, which are able to develop bioactive interfaces with the contacting tissues. These are focused on the prevention of bacterial adhesion and biofilm formation [3] on the implant surface, leading to peri-implant disease and implant failure. Therefore, many studies are concerned with modifying the implant roughness by electrochemical [4], acid etching [5,6] and various blasting procedures [7,8]. The resulting rough surfaces are beneficial for faster healing, cell adhesion, and the osteogenesis process [9]. Similar advances can be achieved by the chemical modification of the titanium surface, leading to a wide range of bioactive coatings, such calcium phosphate ceramics, especially hydroxyapatite [10], titania [11] silica [12,13], zirconia [14], zinc oxide [15], and their composites/mixtures [16].

A great number of self-disinfecting inorganic coatings for titanium implants are based on metal and oxide nanoparticles, which are activated by contact with the appropriate media or by light irradiation [17]. An example of such a coating is a Ag–TiO_2_ layer, which was shown to be active under visible light against the vesicular stomatitis virus [18]. These approaches complete conventional medical procedures in terms of decontaminating the implanted materials and protecting the injured tissues from bacterial colonization, which is common after implantation procedures [19]. According to the literature data, the main mechanisms exhibited by the inorganic nanomaterials to induce pathogen extinction are related to the release of metallic ions, electrostatic interaction with the bacteria membrane, and generating reactive oxygen species (singlet oxygen, superoxide anion, hydroxyl radical, hydrogen peroxide) (ROS) [20]. Photogenerated ROS were investigated as a potential solution in the recent pandemic context due to their wide antimicrobial effect, even against bacterial biofilms and viruses [17].

This work investigates the pathways to developing and characterizing effective titanium coatings appropriate for bioactive dental implants that are able to display self-decontamination prior to implantation through singlet oxygen (^1^O_2_) generation under visible irradiation, antibacterial effect revealed by the interaction with *Micrococcus lysodeicticus* both for inorganic nanostructured layers and the resulting organic/inorganic hybrid systems (Lys/TiO_2_/Ti foil; Lys/Au–TiO_2_/Ti foil, Lys/Ag–TiO_2_/Ti foil) after lysozyme adsorption on the inorganic surface, and biocatalytic effectiveness of the loaded lysozyme for the hydrolysis of a synthetic substrate (4-Methylumbelliferyl β-D-N,N′,N″-triacetylchitotrioside) used previously for monitoring the enzymatic activity of lysozyme after immobilization on solid carriers [21,22].

Moreover, it is important to investigate the engineered TiO_2_-based coatings and their lysozyme loading capacity to better understand the usual in vivo-developed TiO_2_ layer on titanium implants, including their interaction with lysozyme usually found in saliva [23,24]. Although lysozyme is an antibacterial enzyme that is common in nature, present in human body [25], and frequently used as a model protein for fundamental and applicative research studies [26,27] due to its appropriate dimensions and relative stability after immobilization on different solid supports, its action mechanism remains poorly understood.

The aim of the present work was to propose an innovative approach for the dual modification of a sol–gel TiO_2_ layer covering titanium, both with Au/Ag NPs and lysozyme, in order to achieve active nanostructures for safe dental implants with self-disinfecting, antibacterial, and biocatalytic features.

## 2. Materials and Methods

### Material Synthesis

**Development of bare and metal-modified TiO_2_ coatings (Au–TiO_2_/Ti foil, Ag–TiO_2_/Ti foil, TiO_2_/Ti foil)**

Titania precursor sol was prepared according to our previous work [28], using titanium isopropoxide (97% Aldrich), isopropyl alcohol (99.7% Lachner), and 2,4-Pentadione (99% Alfa Aesar), cast by spin coating on Ti foil (0.1 mm thickness, Goodfellow Metals, metal plate samples of 0.7 × 1.5 cm^2^) previously subjected to etching with nitric acid (65% Lachner) for 1 h. In this sense, five successive depositions were made using 10 µL of the synthesized sol subjected to 500 rpm for 60 s (VTC 100 PA, Vacuum Spin Coater, MTI Corporation, UK). After five deposition cycles, thermal treatment in air at 250 °C for 3 h was performed. Aqueous solution (7.5 µL, 3 mM) of gold chloride trihydrate (MP Biomedicals, LLC) or aqueous solution (7.5 µL, 3 mM) of silver nitrate (99.9% Wako) was further added by drop casting on the above-mentioned coatings, which were dried at 80 °C and subsequently treated at 400 °C for 3 h.

**Lysozyme adsorption on inorganic coatings**

The coated metal plates (TiO_2_/Ti, Au–TiO_2_/Ti, Ag–TiO_2_/Ti) were introduced into 4 mL potassium phosphate buffer (PBS, pH 6.5) containing lysozyme (0.4 mg/mL) and gently shaken for 1 h at 25 °C. After removing the supernatant, the plates were washed twice (with PBS and ultrapure water) and dried in a vacuum. Lysozyme (from chicken egg white) and potassium phosphate buffer were contained using a Lysozyme Activity Kit (LY0100) (Sigma Aldrich).

**Scanning Electron Microscopy (SEM)**

SEM images were obtained in a high0resolution microscope (FEI Quanta 3D FEG model) equipped with the Octane Elect X-ray EDS system, in a high vacuum, using an acceleration voltage of 30 kV for both SEM (secondary electrons detection mode) and EDS measurements. NPs size distribution of Au and Ag was estimated with an SPIP (Scanning Probe Image Processor, v. 4.6.0).

**Atomic Force Microscopy (AFM)**

Atomic force microscopy (AFM) measurements were performed with an XE–100 from Park Systems, equipped with XY/Z decoupled scanners, by selecting the “non-contact” mode. This working mode was preferred due to the minimization of tip-sample interaction. All AFM measurements were performed with NCHR tips produced by Nanosensors, with a typical radius of curvature of ~8 nm, a length of ~125 µm, a width of 30 µm, an elasticity constant of ~42 N/m, and a resonance frequency of ~330 kHz. The AFM images were processed with the XEI program (v 1.8.0), produced by the same company (Park Systems).

**X-ray diffraction (XRD)**

The measurements were performed using the Rigaku Ultima IV equipment, with Cu K_α_ radiation and a fixed power source (40 kV and 30 mA). The diffractometer was set in the grazing incidence X-ray diffraction (GIXD) condition with the fixed incidence angle set at α = 0.5°. The films were scanned at a rate of 1°/min over a range of 2θ = 20–90°.

**UV–Vis Spectroscopy (UV–Vis)**

Diffuse reflectance UV–Vis spectra were recorded with a Perkin Elmer Lambda 35 spectrophotometer with a spectral range of 200–1000 nm. The registered reflectance data were transformed into absorption spectra using the Kubelka–Munk function.

**Photoluminescence Spectroscopy (PL)**

Photoluminescence data were registered with a Carry Eclipse fluorescence spectrometer (Agilent Technologies) equipped with thin film accessories. The working parameters were as follows: a scan rate of 120 nm min^−1^, slits were set at 20 nm both in excitation and emission, and measurements were performed at room temperature for λ_exc_ = 270 nm.

**ROS (singlet oxygen ^1^O_2_) identification**

Measurements for singlet oxygen identification were performed in quartz cuvettes containing methanolic solution (5 µM) of SOSG (Singlet Oxygen Sensor Green–Thermo Fisher Scientific/Invitrogen). The interest sample was also placed in the cuvette with the coated side in front of a solar simulator (PECEL equipped with a cut of filter for λ > 420 nm) in order to expose the oxygen singlet (^1^O_2_) under light. Due to its reaction with SOSG (anthracene component), endoperoxide formation occurred. Its presence was further evidenced by a photoluminescence signal peaked around 530 nm for λ_exc_ = 488 nm at every 10 min after light exposure.

**Antibacterial activity of inorganic coatings** (TiO_2_/Ti, Ag–TiO_2_/Ti, Au–TiO_2_/Ti) was tested against *Micrococcus lysodeicticus*.

The samples of interest (0.3 × 0.3 cm^2^) were introduced into 1 mL LB medium and 30 µL *Micrococcus (M.) lysodeikticus* ATCC 4698 cell suspension 0.01% *w/v* in potassium phosphate buffer (Lysozyme Activity Kit –LY0100 Sigma Aldrich) and incubated for 24 h at 37 °C. In order to determine the cell growth, the optical density (OD) at 600 nm was recorded using the multireader Bio Tek Sybergy HTX, Agilent US. The determination of OD values represents a rapid method for microbial cell quantification. All tests were performed in triplicate.

**Lysozyme (Lys/TiO_2_/Ti, Lys/Ag–TiO_2_/Ti, Lys/Au–TiO_2_/Ti) activity assays on microbial substrate (*Micrococcus lysodeicticus*)**

The samples of interest (0.3 × 0.3 cm^2^) were introduced into 1 mL *M. lysodeikticus* cell suspension (ATCC 4698 from Lysozyme Activity Kit, LY0100 Sigma Aldrich, 0.01% *w*/*v* in potassium phosphate buffer) and incubated at 25 °C. The decrease in absorbance at 450 nm was monitored at 5, 10 min, 1 h, and 24 h with the multireader Bio Tek Sybergy HTX, Agilent US. The recorded results were compared with the *M. lysodeikticus* cell suspension (negative control) and lysozyme solution (300 U/mL, positive control). All tests were performed in triplicate.

**Lysozyme (Lys/TiO_2_/Ti, Lys/Ag–TiO_2_/Ti, Lys/Au–TiO_2_/Ti) activity assays on synthetic substrate [4-MU-β-(GlcNAc)_3_]**

Biocatalytic activity of hybrid systems (Lys/TiO_2_/Ti, Lys/Ag–TiO_2_/Ti, Lys/Au–TiO_2_/Ti) was tested for 4-methylumbelliferyl β-D-N,N′,N″-triacetylchitotrioside [4-MU-β-(GlcNAc)_3_] hydrolysis reaction. The formation of a fluorescent reaction product, namely, 7-hydroxy-4-metyl coumarin was monitored by spectroscopy fluorescence measurements, with a PL emission signal being registered at 450 nm for λ_exc_ = 355 nm.

In order to perform the enzymatic assay, hybrid organic/inorganic samples (0.7 × 1.5 cm^2^) were kept for 3 h at 30 °C in 3 mL buffered solution of 4-MU-β-(GlcNAc)_3_ (0.01 mg/mL, pH 7). The release in the solution of the fluorescent reaction product was proved by photoluminescence measurements for λ_exc_ = 355 nm, with an emission peak centered at 447 nm being present. The measurements were conducted with a Carry Eclipse fluorescence spectrometer (Agilent Technologies). The enzymatic activity tests were performed in triplicate.

## 3. Results

### 3.1. SEM

SEM investigations allow the Au- and Ag-modified TiO_2_ films covering the Ti foil to be explored.

**Figure 1:**
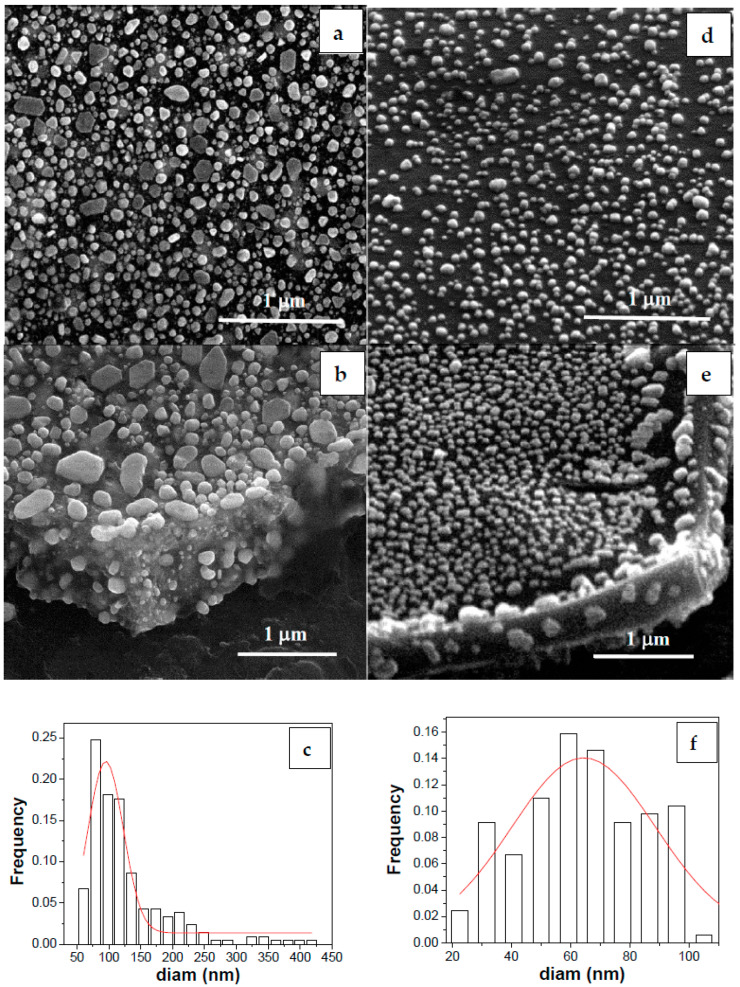
SEM micrographs of Au–TiO_2_ film (**a**,**b**) and Ag–TiO_2_ film (**d**,**e**), showing surface Au top view (**a**) and Ag tilted view (**d**) together with cross-section edge views (**b**,**e**) of the films. The particle size distribution for Au is presented in (**c**) while the Ag NPs histogram distribution is shown in (**f**).

The microstructural study by SEM revealed the formation of a titania film with Au-faceted particles, from 30 to 400 nm in size (Figure 1a,b). The cross-section image (Figure 1b) of the Au–TiO_2_ film showed the presence of Au particles embedded inside the film, with a uniform distribution. SEM micrographs of the Ag–TiO_2_ film (Figure 1d,e) showed the presence of Ag nanoparticles with relatively uniform sizes, i.e., ~50–100 nm, at the film surface (Figure 1d), while the cross-section (Figure 1e) at the film edge showed aggregates of a few nanoparticles embedded inside the film. The NPs size distribution of the Au (Figure 1c) and Ag (Figure 1f) showed that the Au NPs exhibited a larger size (with the majority centered on 100 nm) in comparison with Ag NPs, with an average size of ~60 nm.

In addition, from Figure 1b,e, the substrate for the TiO_2_ layer (bottom right corner) can be observed, namely, the titanium foil exposing a rough and defective surface. This is due to the treatment with nitric acid, which was meant to increase the adhesion of the sol-containing titanium precursor. According to the literature [29], the acid etching procedure is also used to decontaminate the implant surface. Au and Ag nanoparticles cover the titania surface and can also be identified in the layer thickness, displaying a composite structure. The two-stage thermal treatment applied to the investigated coatings was responsible for the nanoparticle mixture, i.e., the 250 °C air treatment of the Ti foil coated with the titanium containing sol produced an amorphous structure. This was converted into a crystalline phase (anatase) at the second stage of thermal treatment (at 450 °C) also involving the metal precursors.

**Figure 2:**
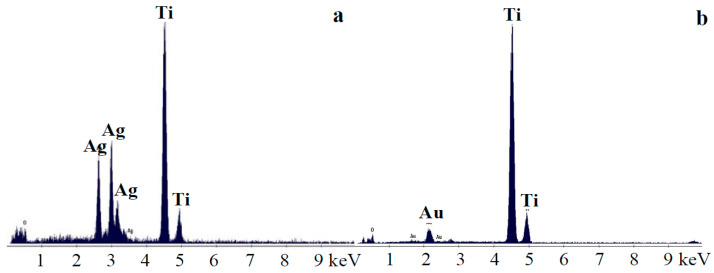
EDS spectra of the Ag–TiO_2_ film (**a**) and Au–TiO_2_ film (**b**).

Chemical elemental analysis by EDS (Figure 2) confirmed the presence of Au and Ag particles decorating the titania films. Taking into account the atomic weights of Ag, Au, O, and Ti, the EDS-calculated Ag/TiO_2_ volume ratio was found to be ~30 +/− 8%, while for the Au/TiO_2_, the evaluated volume ratio was ~12 +/− 5%. In fact, the functional “fingerprint” of the metallic nanoparticles dispersed on the titania layer surface should be further explored in terms of the antibacterial activity of the synthesized coatings, a hindering effect of bacterial growth being presumable. The volume ratio of the metal particles and the TiO_2_ matrix was evaluated from EDS measurements.

### 3.2. AFM

**Figure 3:**
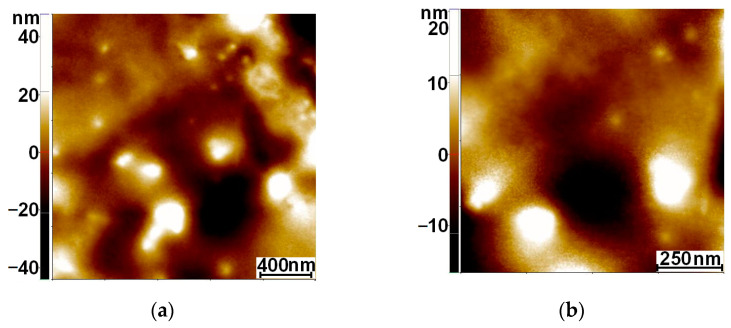

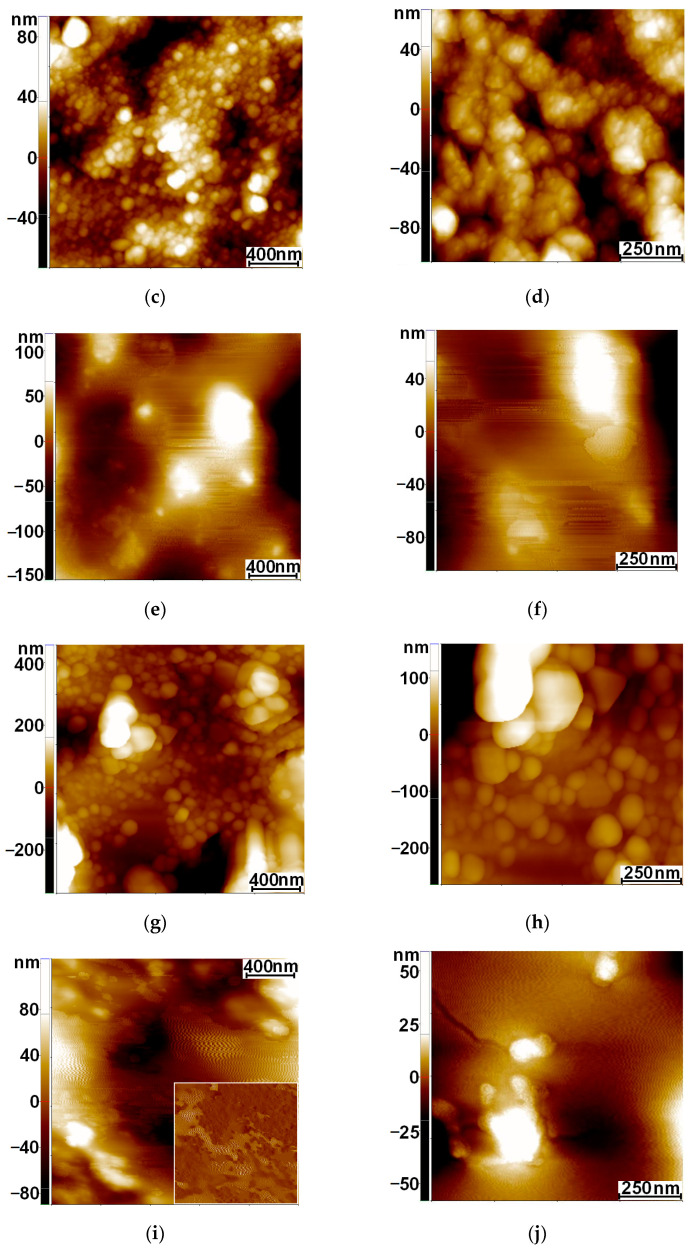

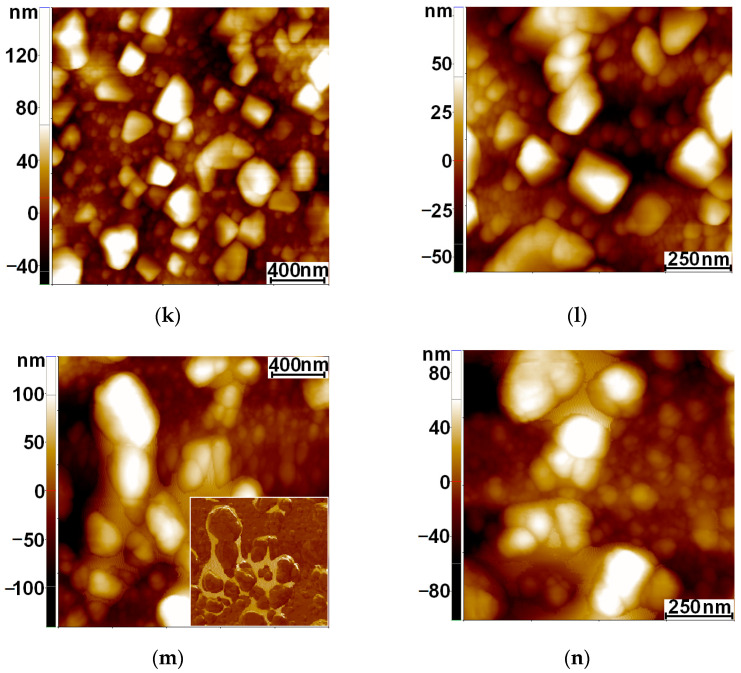
2D AFM images at a scale of 2 × 2 µm^2^ (**left** column) and 1 × 1 µm^2^ (**right** column) for bare Ti foil (**a**,**b**), TiO_2_/Ti (**c**,**d**), Lys/TiO_2_ (**e**,**f**), Au-modified TiO_2_ (**g**,**h**), Lys/Au–TiO_2_/Ti (**i**,**j**), Ag-modified TiO_2_ (**k**,**l**) and Lys/Ag-modified TiO_2_ films (**m**,**n**). Phase contrast 2D AFM images are superimposed for Lys/Au/TiO_2_/Ti in (**i**) and for Lys/Ag/TiO_2_/Ti in (**m**).

Figure 3 comparatively presents the morphology of all investigated samples by AFM at the scales of 2 × 2 µm^2^ (left column) and 1 × 1 µm^2^ (right column). Thus, Figure 3a,b show the topography of the bare Ti foil used as the substrate. The bare Ti substrate exhibits some irregularities consisting of pits and valleys, most probably formed during the nitric acid treatment.

The root mean square roughness (R_q_) at the scale of 2 × 2 µm^2^ was found to be 10.8 nm, and the peak-to-valley parameter (R_pv_), which is the height between the lowest and the highest points on the scanned area, was 92.0 nm;. At the scale of 1 × 1 µm^2^, they reached 5.6 nm (R_q_) and 36.8 nm (R_pv_), respectively. Figure 3c,d present 2D AFM images of the Ti foil (2 × 2 and 1 × 1 µm^2^) covered by TiO_2_. The morphology of the surface consists of quasi-spherical shaped particles ~20 nm in diameter (random agglomerated particles/clusters can also be observed). At the scale of 2 × 2 µm^2^, the following corrugation parameters were observed: R_q_ = 21.3 nm and R_pv_ = 169.1 nm, while at the scale of 1 × 1 µm^2^, they were R_q_ = 19.1 nm and R_pv_ = 166.4 nm. After covering the TiO_2_ surface with lysozyme (Figure 3e,f), a high adhesion to the TiO_2_ film was observed. The surface became completely covered with the enzymatic layer, with the TiO_2_ particles becoming almost unrecognizable below the lysozyme layer. For the Lys/TiO_2_/Ti sample, the following corrugation parameters were observed: R_q_ = 33.8 nm and R_pv_ = 272.4 nm at the scale of 2 × 2 µm^2^, while at the scale of 1 × 1 µm^2^, they were R_q_ = 27.3 nm and R_pv_ = 182.0 nm.

Furthermore, the surface modification with noble metal NPs led to a good dispersion of Au (Figure 3g,h) and Ag NPs (Figure 3k,l) on the TiO_2_/Ti films. The line scans collected in the fast-scan direction (not shown here) express a similar height in the vertical direction of ~250 nm (from −150 to 100 nm) for both samples. The root mean square (RMS) roughness, R_q_, of the Au-modified TiO_2_ sample, at the scale of 2 × 2 µm^2^, was ~82.1 nm, and the peak-to-valley parameter, R_pv_ reached 793.7 nm. At the 1 × 1 µm^2^, they were R_q_ = 57.5 nm and R_pv_ = 424.4 nm. Meanwhile, for the Ag-modified TiO_2_ sample, at the scale of 2 × 2 µm^2^, R_q_ equaled 28.4 nm and R_pv_ ~210.4 nm, while at the 1 × 1 µm^2^ scale, they were R_q_ = 22.1 nm and R_pv_ = 137.3 nm.

The samples were further loaded with lysozyme and the resulting AFM images are presented in Figure 3i,j for the Lys/Au–TiO_2_/Ti sample and in Figure 3m,n for the Lys/Ag–TiO_2_/Ti sample. The images at larger scales suggested that the lysozyme loading capacity was higher for the Au-modified TiO_2_ sample, since the corresponding image appears less clear and has “noisy” areas (see for example the phase contrast image superimposed in the bottom right corner of Figure 3i).

On the other hand, the phase contrast AFM image superimposed on the topographical one in Figure 3m suggests that lysozyme was preferentially located/agglomerated at the grain boundaries of Ag NPs, while on Au-modified TiO_2_, it covered uniformly in larger parcels. The corrugation parameters, for lysozyme-loaded samples, at the scale of 2 × 2 µm^2^, were found to be 43.0 nm (R_q_) and 286.7 nm (R_pv_) for Lys/Au-modified TiO_2_, and 50.2 (R_q_) and 278.0 nm (R_pv_), respectively, for Lys/Ag-modified TiO_2_. At the scale of 1 × 1 µm^2^, they were R_q_ = 10.3 nm and R_pv_ = 117.0 nm for Lys/Au-modified TiO_2_, while they were R_q_ = 50.1 nm and R_pv_ = 277.9 nm for Lys/Ag-modified TiO_2_, demonstrating that the lysozyme exhibits a local smoothing tendency for Au as compared with Ag-modified TiO_2_.

The following aspects can be summarized from the AFM analysis:

(i) The Ti foil has a defective or irregular surface; (ii) TiO_2_ nanoparticles are gathered forming dense layers; (iii) metal NPs are well dispersed and faceted; (iv) lysozyme covers the upper surface in the following order: TiO_2_/Ti > Au–TiO_2_/Ti > Ag–TiO_2_/Ti; (v) the relatively large roughness parameter values are favorable towards the adhesion of biological compounds.

### 3.3. XRD

The crystallinity of the films was studied using the X-Ray diffraction (XRD) method. The mean size of the ordered (crystalline) domains, L (commonly known as crystallite sizes), of the phases was calculated using the Scherrer equation: L = Kλ/βcosθ, where K is a dimensionless shape factor, usually taken as 0.89; λ (nm) is the wavelength of XRD radiation (Cu); β (in radians) is the line broadening at half the maximum intensity (FWHM); and θ is the Bragg angle.

**Figure 4:**
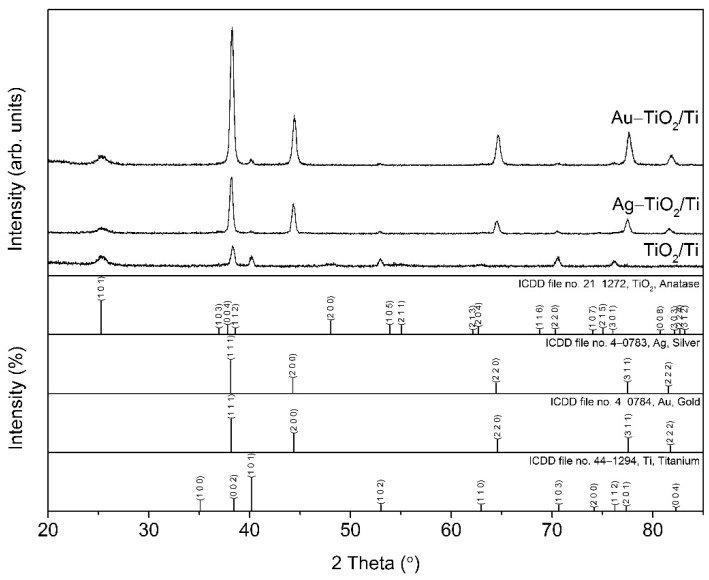
XRD diffractograms of the investigated samples: Au–TiO_2_/Ti, Ag–TiO_2_/Ti, TiO_2_/Ti.

Figure 4 shows the X-Ray diffraction patterns of the TiO_2_/Ti, Ag–TiO_2_/Ti, and Au–TiO_2_/Ti samples. The TiO_2_/Ti sample contained anatase, TiO_2_, and metal titanium, Ti. The reflection of metal titanium phase, Ti, identified according to ICDD file no. 44–1294, belonged to the substrate. A preferred orientation along the (002) crystal plane was observed. The anatase phase, identified according to ICDD file no. 21–1272, only presented a few broad reflections, at 2θ values of 25.26°, 48.08°, 55.04°, and 62.66°, respectively, for the (101), (200), (105) + (211), and (204) crystal planes. The crystallite size of anatase, which was only calculated for the (101) crystal plane, was around 8 nm. Both the Ag–TiO_2_/Ti and Au–TiO_2_/Ti samples contained anatase, TiO_2_, and titanium, Ti, from the metal substrate. In the Ag–TiO_2_/Ti sample, well crystalized silver, Ag, with narrow diffraction lines, was identified according to ICDD file no. 4–0783. The crystallite size for silver was around 23 nm. In the Au–TiO_2_/Ti sample, gold, Au, was detected according to ICDD file no. 4–0784, respectively. The gold phase was very well crystalized, with narrow diffraction lines. The crystallite size for the gold was around 25 nm.

### 3.4. UV–Vis Spectroscopy

**Figure 5:**
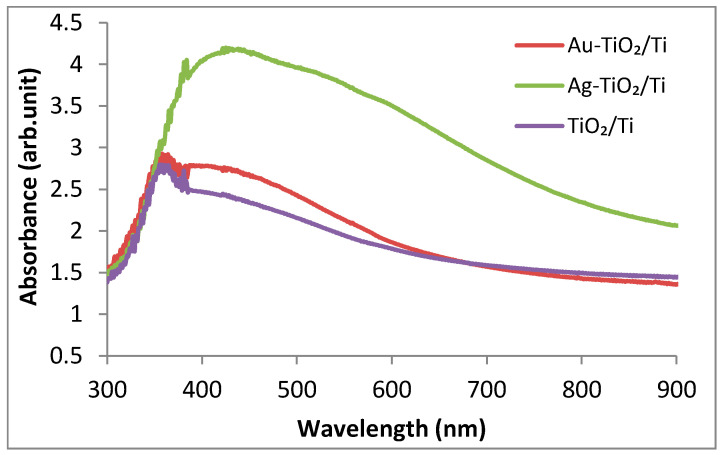
UV–Vis spectra of the Au–TiO_2_/Ti, Ag–TiO_2_/Ti, TiO_2_/Ti samples ranging in the 300–900 nm domain.

In order to explore the light sensitivity of the investigated samples, UV–Vis spectra were recorded. For the Au–TiO_2_/Ti and TiO_2_/Ti samples, Figure 5 reveals the UV light absorption around 370 nm due to the TiO_2_ contribution. In the case of Ag–TiO_2_/Ti, this appears to be scarcely defined and red shifted, merging with a large absorption band spanning from 390 to 800 nm.

Au–TiO_2_/Ti also displayed an absorption band between 390 and 600 nm, which was less intense than that of the silver-containing sample, indicating the presence of Au nanoparticles but also defects in the TiO_2_ nanostructured layer. These led to a long tail in the visible range for the TiO_2_/Ti sample.

The literature concerning Ag-modified TiO_2_ correlates the broad plasmonic peak with the presence of Ag nanoparticles with a large size distribution [30].

### 3.5. Photoluminescence Measurements

**Figure 6:**
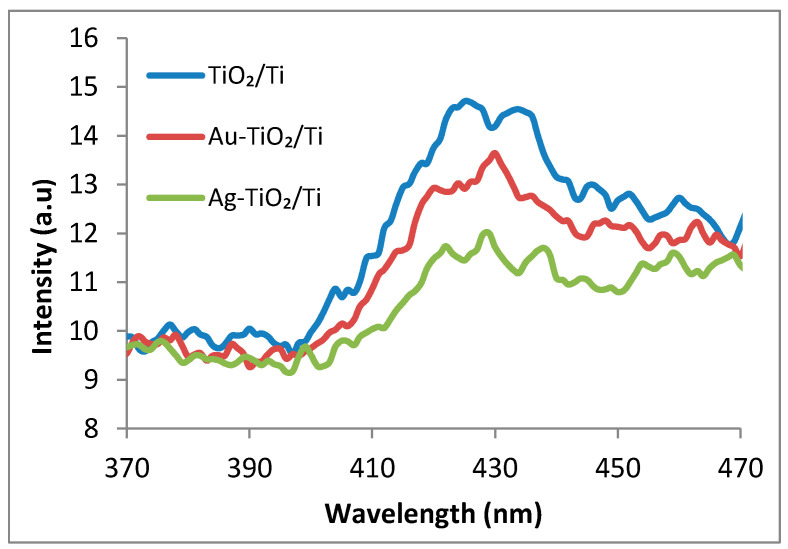
Photoluminescence spectra of TiO_2_/Ti, Au–TiO_2_/Ti and Ag–TiO_2_/Ti samples registered for λ_exc_ = 270 nm.

Generally, the photoluminescent signal of a semiconductor nanomaterial is associated with recombination of the photogenerated electron–hole pairs [31], with significant variations being induced by the modifiers. As TiO_2_ is a well-known nontoxic photocatalyst, many pathways have been explored to improve its light sensitivity, especially in the visible range, including the deposition of noble metal nanoparticles. Usually, for optimal-ratio NPs/TiO_2_, the PL emission decreases relative to the bare TiO_2_ [32], with the recombination of photoinduced charge carriers being hindered by the electron transfer from TiO_2_ to metal. From Figure 6, a broad band ranging from 400 to 470 nm with maximum located at 426 nm can be observed. For TiO_2_ and Au, Ag NPs modified-TiO_2_ samples, Chen et al. [33] assigned a PL signal peak at 417 nm to free excitons, with photoluminescence quenching also being observed for metal-modified materials. In Figure 6, a small PL decrease for Au–TiO_2_/Ti and Ag–TiO_2_/Ti can be perceived, the existence of an active interface between the metal and semiconductor being expected.

### 3.6. ROS (Singlet Oxygen ^1^O_2_) Identification

Nowadays, the use of singlet oxygen in photodynamic therapy is a focus of investigation [34,35], as it is a promising tool against antibiotic-resistant bacteria. There are various photosensitizers used for singlet oxygen generation, such as porphyrins and noble metal nanoparticles.

**Figure 7:**
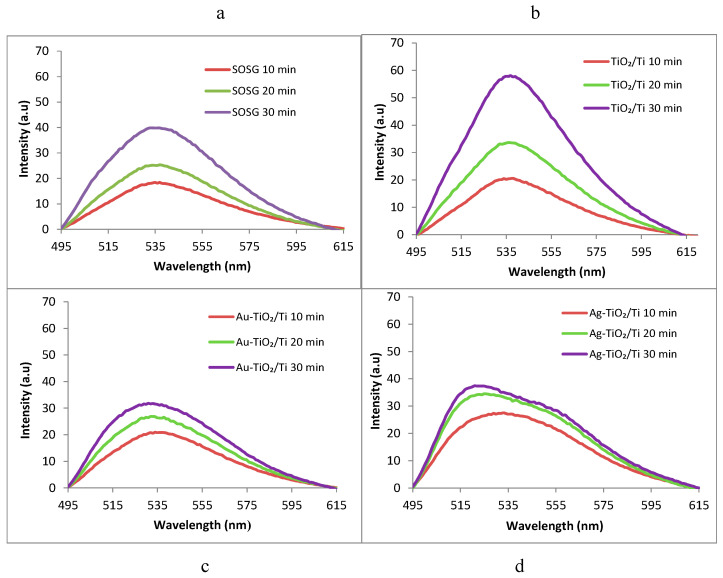
Time course of singlet oxygen formation after exposure to visible light (λ > 420 nm) in the absence (**a**)/presence (**b**–**d**) of the investigated samples, registered with SOSG singlet oxygen sensor for λ_exc_ = 488 nm.

According to our previous work and the literature [36], singlet oxygen (^1^O_2_) is photogenerated by the target samples exposed to light irradiation and further triggers endoperoxide formation from the anthracene component of SOSG green reagent [37], which generates a PL emission peak centered at 535 nm for λ_exc_ = 488 nm. Figure 7 shows the intensity of the PL signals increasing with light exposure, peaking at 536 nm for both blank SOSG (a) and the samples of interest (b), (c), (d). However, higher peaks can clearly be observed for the bare TiO_2_/Ti sample (b), with the metal-modified examples (c,d) generating similar signals as the blank SOSG (a small self-decomposition rate and singlet oxygen production from SOSG itself was previously reported [36,38]). Accordingly, the ability of the TiO_2_/Ti sample to produce singlet oxygen under visible light exposure and its self-decontaminating behavior were verified.

### 3.7. Antibacterial Activity Assays of Inorganic Coatings TiO_2_/Ti, Ag–TiO_2_/Ti, and Au–TiO_2_/Ti against M. lysodeicticus

**Figure 8:**
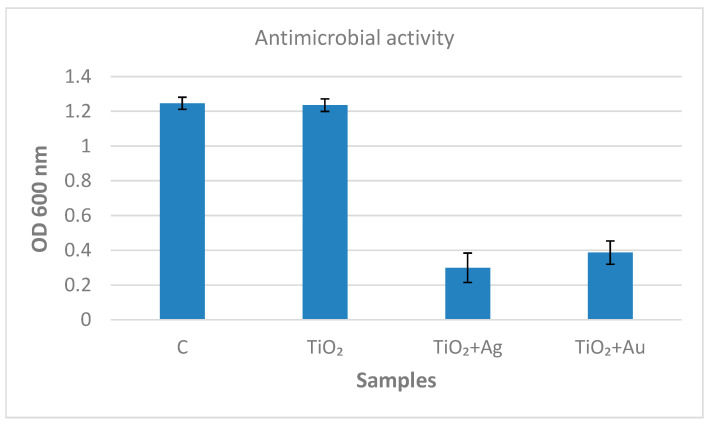
Microbial growth over the TiO_2_-based coatings of titanium.

Figure 8 clearly demonstrates the metal-modified coatings’, i.e., Ag–TiO_2_/Ti and Au–TiO_2_/Ti, significant antimicrobial activity against *M. lysodeicticus*. The cellular viability registered for the metal-modified samples was 24% for Ag and 31% for Au modifiers. By comparison with the control sample (the C microbial cells alone), the bare TiO_2_/Ti exhibited no antimicrobial activity. According to these results, the noble metal nanoparticles added to the TiO_2_ layer hindered microbial growth.

### 3.8. Lysozyme (Lys/TiO_2_/Ti, Lys/Ag–TiO_2_/Ti, Lys/Au–TiO_2_/Ti) Activity Assays on Microbial Substrate (Micrococcus lysodeicticus)

*M. lysodeicticus* cell lysis in the presence of the newly developed hybrid systems was performed.

**Figure 9:**
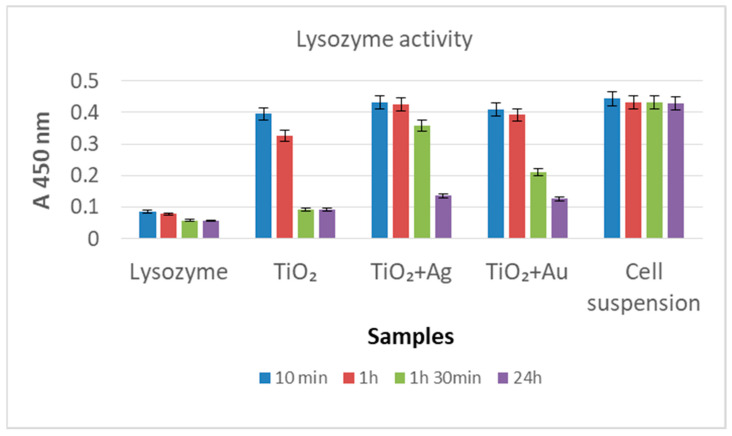
Activity of lysozyme (free and loaded on inorganic coatings) against *M. lysodeicticus*.

The decrease in absorbance at 450 nm measured for the *M. lysodeicticus* suspension in contact with the investigated samples allowed us to evaluate their bioactivity. Accordingly, Figure 9 shows major lysis of *M. lysodeikticus* cells in the first 10 min for the control sample (the free lysozyme in the *M. lysodeicticus* suspension) and a slight advancement during the next 24 h. A similar result was obtained for the Lys/TiO_2_/Ti sample but for a longer incubation time (24 h).

The loaded lysozyme appears to have a slower reactivity than the free enzyme, but this is significant and clearly evidenced after 24 h (as compared with the blank test carried out with the *M. lysodeikticus* cell suspension alone). Based on Figure 9, the following overall activity sequence for the target samples can be proposed: Lys/TiO_2_/Ti > Lys/Au–TiO_2_/Ti > Lys/Ag–TiO_2_/Ti.

### 3.9. Lysozyme (Lys/TiO_2_/Ti, Lys/Ag–TiO_2_/Ti, Lys/Au–TiO_2_/Ti) Activity Assays on Synthetic Substrate [4–MU–β– (GlcNAc)_3_]

The biocatalytic assays described herein were performed in the presence of hybrid systems (Lys/TiO_2_/Ti, Lys/Ag–TiO_2_/Ti, Lys/Au–TiO_2_/Ti) using a synthetic substrate, namely, 4-Methylumbelliferyl β-D-N,N′,N″-triacetylchitotrioside [4-MU-β-(GlcNAc)_3_]. In order to evaluate the hydrolytic capacity of the loaded lysozyme, the formation of a fluorescent reaction product, namely, 7-hydroxy-4-metylcoumarin (4-methylumbelliferone), was monitored by fluorescence spectroscopy, according to previously reported data [21,22,39].

**Figure 10:**
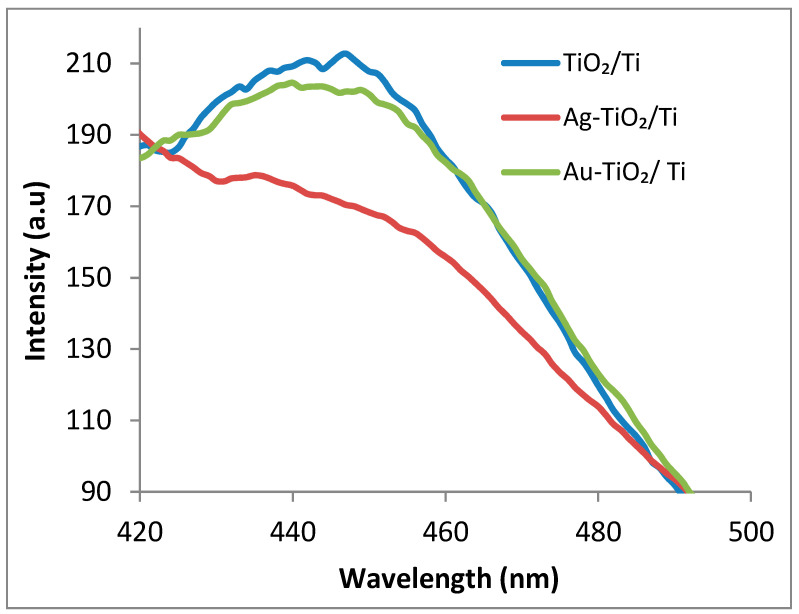
Fluorescence spectra of the released 7-hydroxy-4-metylcoumarin in buffer solution (λ_exc_ = 355 nm, λ_em_ = 450 nm) after 3 h of reaction.

Figure 10 displays emission peaks with maxima at 450 nm assigned to the fluorescent compound 4-methylumbelliferone. This results from the hydrolysis reaction of the buffered organic substrate [4-MU-β-(GlcNAc)_3_] subjected to incubation at 37 °C for 3 h in the presence of the previously prepared hybrid systems. Only Lys/TiO_2_/Ti and Lys/Au–TiO_2_/Ti exhibited well-defined peaks. Accordingly, for these inorganic coatings, a significant lysozyme loading capacity, the preservation of enzymatic activity after immobilization, and the release of fluorescent product into the buffer solution can be assumed. Unlike the above-mentioned hybrid systems, the Ag-containing sample produced insignificant amounts of 4-methylumbelliferone. Based on Figure 10, the resulting activity sequence for the samples of interest is as follows: Lys/TiO_2_/Ti > Lys/Au–TiO_2_/Ti > Lys/Ag–TiO_2_/Ti.

## 4. Discussion

The development and structural characterization of bare and metal-modified TiO_2_ coatings, appropriate for titanium dental implants, is an important area of study for engineered nanomaterials for biomedical applications. The present approach intended to simultaneously identify and test the key parameters required to produce safe and functional coatings for titanium implants, keeping in mind the natural processes triggered by the implant’s presence, e.g., the formation of a thin TiO_2_ layer on the titanium surface, bacterial colonization, and the activity of the lysozyme present in saliva. Therefore, it is important to understand the reactivity of the TiO_2_ thin film on titanium relative to the contact environment, and to optimize the structural and functional parameters to a trigger self-disinfecting ability, intrinsic antibacterial properties, and the properties induced by the lysozyme loading capacity. In order to enhance the photosensitivity and antimicrobial properties of TiO_2_, the deposition of noble metal nanoparticles was successfully performed.

SEM micrographs (Figure 1) show a sol–gel TiO_2_ layer covering the titanium foil, decorated with Au/Ag nanoparticles (Figure 2, EDS spectra). The XRD analysis confirmed its anatase structure and the crystalline state of the modifiers used. Spectroscopic measurements revealed the improvement of TiO_2_ light absorption after metal modification (UV–Vis). Generally, for gold nanoparticles, the data from the literature show strong plasmon resonance absorption [40]. This appears to be dependent on several light absorbing material features, such as shape, the size distribution of metallic nanoparticles, interaction between particles, and the dielectric environment [41]. In the present study, a broad absorption band was present for the Ag-TiO_2_/Ti sample, which strongly decreased for the Au–TiO_2_/Ti sample. This may be related to the large particle size distribution of the metallic nanoparticles. The gold and silver crystallite size identified by XRD was around 25 and 23 nm, respectively. However, SEM and AFM measurements revealed bigger, faceted surface particles. For these crystallite aggregates, a preferential growth in the (111) direction was noticed according to the main diffraction peak.

The AFM investigation also revealed the lysozyme interaction with the surface of the investigated samples. The recorded images indicated a different lysozyme coverage. This is in line with the enzymatic activity sequence of the newly developed hybrid systems.

Radical trapping measurements demonstrated singlet oxygen generation under visible light irradiation for the TiO_2_/Ti sample.

By comparing the Figure 8, Figure 9 and Figure 10, a complementary antimicrobial mechanism can be observed for the investigated materials: (a) Au and Ag nanoparticles deposited on TiO_2_ trigger the antimicrobial effect of the inorganic coatings; (b) the adsorbed lysozyme, especially on bare TiO_2_, preserves its enzymatic activity and could provide antibacterial protection for dental implants.

These experimental results are important since many studies are devoted to developing and improving nonaggressive antimicrobial tools, including the activity of enzymes [42]. In this sense, the key role of lysozyme in human immune defense is well recognized and has been studied; however, its mechanism of action is not fully understood. Ibrahim et al. [43] distinguish between lysozyme’s bactericidal activity and its catalytic function. Therefore, further investigations on its bioactivity are needed together with the development of lysozyme-based hybrid materials.

## Data Availability

All data were reported in the paper.
